# Association Between Endogenous Equol Production and the Onset of Overactive Bladder in Postmenopausal Women

**DOI:** 10.3390/jcm14124183

**Published:** 2025-06-12

**Authors:** Hiroyuki Honda, Tomohiro Matsuo, Hidenori Ito, Shota Kakita, Shintaro Mori, Kyohei Araki, Kensuke Mitsunari, Kojiro Ohba, Yasushi Mochizuki, Ryoichi Imamura

**Affiliations:** Department of Urology, Graduate School of Biomedical Sciences, Nagasaki University, Nagasaki 852-8501, Japan; h.honda2306@nagasaki-u.ac.jp (H.H.); myselfhide0x0@yahoo.co.jp (H.I.); s-kakita@nagasaki-u.ac.jp (S.K.); s-mori@nagasaki-u.ac.jp (S.M.); araki.k@nagasaki-u.ac.jp (K.A.); kmitsunari@nagasaki-u.ac.jp (K.M.); ohba-k@nagasaki-u.ac.jp (K.O.); mochi@nagasaki-u.ac.jp (Y.M.); ryo-imamura@nagasaki-u.ac.jp (R.I.)

**Keywords:** equol, women, menopause, overactive bladder, lower urinary tract symptom

## Abstract

**Objectives**: Equol, a gut-derived metabolite of soy isoflavones with estrogenic activity, may influence bladder aging. However, the association between overactive bladder (OAB), which is closely linked to bladder aging, and the estrogenic effects of equol remains unknown. Therefore, this study investigated the association between endogenous equol production and onset and severity of OAB in postmenopausal women. Methods: The study included 128 postmenopausal women, newly diagnosed with OAB, who were categorized into equol- and non-equol-producing groups based on urinary equol levels as measured by enzyme-linked immunosorbent assay. Patient clinical characteristics, OAB Symptom Score (OABSS), and urodynamic parameters were assessed. Propensity score matching was performed to minimize confounding factors related to the timing of lower urinary tract symptom (LUTS) onset. Results: Equol producers exhibited a significantly later onset of LUTS than non-producers (68.7 ± 10.9 vs. 62.7 ± 10.7 years, *p* = 0.002). Equol producers were more prevalent in the late-onset group (58.6% vs. 31.0%, *p* = 0.002), which had significantly higher urinary equol concentrations than the early-onset group (*p* = 0.014). No significant differences were observed in total OABSS or subscale scores between the groups, suggesting that equol did not affect symptom severity. Propensity score-matched analysis (n = 104) confirmed that equol non-production was an independent risk factor for early-onset LUTS (OR, 1.930; 95% CI, 1.248–4.049; *p* = 0.014). **Conclusions**: Endogenous equol production was significantly associated with the delayed onset of OAB in postmenopausal women. Thus, equol may serve as a protective factor and non-invasive biomarker to guide individualized prevention and early intervention strategies in urological care for women.

## 1. Introduction

Overactive bladder (OAB) is a symptomatic syndrome characterized by urinary urgency and frequency and is associated with reduced quality of life and healthy life expectancy in affected patients [1,2]. The prevalence of OAB increases with age, affecting 13.8% individuals aged ≥40 years and over 30% of those aged ≥80 years, as reported in a recent epidemiological survey conducted in Japan [3].

In addition to aging, other contributing factors to OAB include lifestyle-related diseases such as hypertension, diabetes mellitus, oxidative stress, and estrogen deficiency. Particularly, estrogen deficiency plays a critical role in reducing elasticity and blood flow in the smooth muscle and connective tissue around the bladder and urethra, and bladder mucosa. Estrogen deficiency reportedly increases detrusor muscle hyperactivity and sensitivity to bladder contractions, leading to urinary frequency and urgency [4]. Furthermore, estrogen deficiency is a recognized risk factor for several lifestyle-related diseases [5] and indirectly contributes to the development of OAB. Accordingly, topical vaginal estrogen therapy has been shown to alleviate OAB symptoms in women with OAB by improving urogenital atrophy [6]. These findings underscore the significant influence of estrogen on the development and treatment of OAB.

The menopausal transition is a critical period in women’s lives, marked by hormonal fluctuations that affect reproductive function and overall physical and mental health. Symptoms such as sleep disturbances, mood changes, and urogenital discomfort often emerge during this stage and may persist for years, significantly impairing daily functioning and quality of life [7]. Therefore, understanding modifiable factors that influence these symptoms is essential for promoting healthy aging in women.

Equol, a phytoestrogen with a structural formula similar to estrogen, exhibits estrogenic effects by binding to estrogen receptors (ERs) [8]. Consequently, several studies have reported the efficacy of equol in managing menopausal disorders and lifestyle-related diseases such as hypertension and diabetes [9,10,11]. However, no study has investigated the association between OAB, which is closely linked to lifestyle diseases, and the estrogenic effects of equol. Therefore, this study aimed to clarify the relationship between equol production and OAB symptoms in women.

## 2. Materials and Methods

### 2.1. Ethics

This study was approved by the Nagasaki University Hospital Ethics Committee (Approval Number: 20122138) and conducted in compliance with the principles outlined in the Declaration of Helsinki. Written informed consent was obtained from all participants before their inclusion in the study.

### 2.2. Patients and Study Design

**Figure 1:**
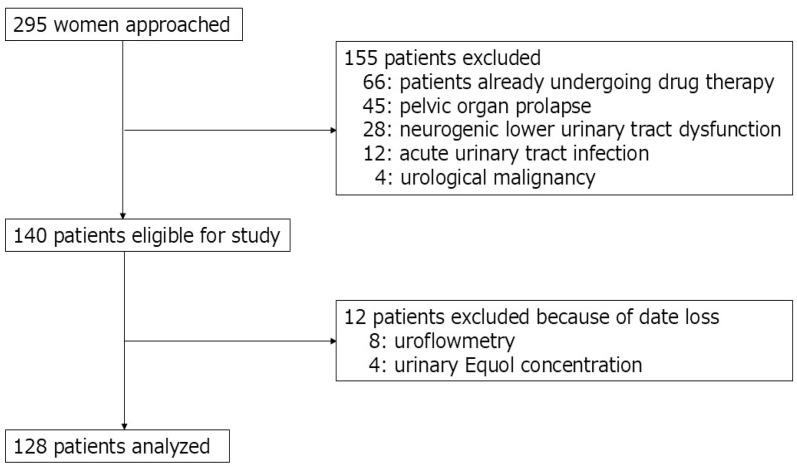
Patient flow diagram.

A total of 128 postmenopausal women were ultimately included in this study. The study initially recruited 295 consecutive women who visited the outpatient urology department at Nagasaki University Hospital between January 2019 and December 2023. Postmenopausal status was defined as the absence of menstruation for at least 12 consecutive months. All included patients were newly diagnosed with OAB based on the Overactive Bladder Symptom Score (OABSS), specifically defined as a score of ≥2 on Question 3 (urgency) and a total score of ≥3 [12]. The exclusion criteria included current treatment for OAB, acute urinary tract infection, pelvic organ prolapse, urological malignancy, or neurogenic lower urinary tract dysfunction (Figure 1). Of the 295 women initially assessed, 167 were excluded according to predefined exclusion criteria.

The patients were divided into two groups based on their ability to produce equol, as determined by using spot urine samples: an equol-producing (P) group and an equol-non-producing (NP) group. Retrospective analyses were conducted to compare patient characteristics and urological parameters between the two groups. Additionally, the patients were categorized into early- and late-onset groups based on the median age at which they first recognized at least one lower urinary tract symptom (LUTS) including OAB. The age at onset was determined through patient interviews conducted during the initial consultation at our hospital. These subgroups were analyzed using the same methods to investigate the differences in characteristics and urological parameters. Subjective symptoms were assessed using the OABSS at the initial visit, where OAB severity was classified as mild (≤5 points), moderate (6–11 points), or severe (≥12 points) based on the total OABSS. Objective findings including voided volume (VV) and maximum flow rate (Qmax) were measured using free uroflowmetry with the AQUARIUS^®^ CTS (Laborie Medical Technologies, Corp., Portsmouth, NH, USA). Post-void residual urine volume (PVR) was measured via ultrasound sonography (ARIETTA 65LE^®^, Fuji Film Healthcare, Tokyo, Japan).

### 2.3. Urinary Sample Preparation and Assessment

Spot early morning urine samples were collected from participants during their visits to our institution to estimate their equol production levels. Urine specimens from all participants were centrifuged at 3000× *g* for 10 min, and the supernatants were separated and stored at −80 °C until analysis. After thawing, urinary equol concentrations were measured using an enzyme-linked immunosorbent assay (ELISA) kit (Equol ELISA Kit^®^; Healthcare Systems Co., Ltd., Aichi, Japan) following the manufacturer’s instructions. Absorbance was measured at 450 nm using a microplate reader (Thermo LabSystems Multiskan RC; Artisan Technology Group, Champaign, IL, USA). The cut-off value for equol production was defined as a urinary equol concentration ≥ 1.0 µmol, based on a previous study [11].

### 2.4. Statistical Analysis

All statistical analyses were conducted using EZR version 1.68 (Saitama Medical Center, Jichi Medical University, Saitama, Japan), which is a graphical user interface for R version 4.3.1 (The R Foundation for Statistical Computing, Vienna, Austria). All statistical data are presented as the mean ± standard deviation or number of patients (percentage, %). The sample size was calculated using G*Power^®^ version 3.1 software, based on previous studies [13,14]. Considering a two-sided significance level of 0.05, power of 80%, and effect size of 0.5, the ideal sample size was calculated to be at least 118 participants, with 59 patients in each group.

The normality of continuous variables was assessed using the Shapiro–Wilk test. Variables that followed a normal distribution were compared between the P and NP groups using Student’s *t*-test, whereas non-normally distributed variables were compared using the Mann–Whitney U test. Categorical variables were compared using the chi-square test. Associations between urinary equol concentration and each parameter were evaluated using Pearson’s correlation coefficient. Univariate analyses were conducted using logistic regression. Statistical significance was set at *p* < 0.05.

### 2.5. Propensity Score Matching

Propensity score matching was used to evaluate whether equol production was associated with the onset of LUTS, including OAB. Thus, early-onset patients with LUTS were matched 1:1 with late-onset individuals based on their propensity scores using nearest-neighbor matching and a caliper width of 0.2 SDs.

## 3. Results

### 3.1. Differences in Patient Characteristics

Table 1 summarizes the patient characteristics. Among 128 eligible patients, 59 (46.1%) and 69 (53.9%) comprised the P and NP groups, respectively. The age when patients visited our institution tended to be higher in the P group than that in the NP group (73.2 ± 9.8 years vs. 70.2 ± 10.6 years; *p* = 0.094). The age of LUTS onset was 68.7 ± 10.9 years for the P group and 62.7 ± 10.7 years for the NP group, with a significantly younger onset age observed in the NP group (*p* = 0.002). The P group (61.0%) had a higher prevalence of hypertension (61.0%) than the NP group (40.6%) (*p* = 0.033). However, no significant differences were observed in other comorbidities, including diabetes, dyslipidemia, and chronic kidney disease, between the two groups (Table 1).

### 3.2. Differences in Urological Parameters

No significant difference was observed in total OABSS as the subjective symptom at the time of OAB diagnosis between the two groups (P group, 8.2 ± 3.0; NP group, 8.6 ± 3.1; *p* = 0.429). In addition, no significant differences were observed in any subscale scores of the OABSS between the two groups. Similarly, the distribution ratios for OAB severity did not differ significantly between groups (*p* = 0.845) (Table 1).

Regarding objective findings, VV and PVR were lower in the P group than in the NP group. Qmax was also lower in the P group; however, the differences were not significant for VV (*p* = 0.231), PVR (*p* = 0.577), or Qmax (*p* = 0.610) (Table 1).

### 3.3. The Relationship Between the Ability to Produce Equol and the Onset Time of LUTS

The median age at which patients first became aware of LUTS, including OAB, was 65.0 years [range, 40–88 years]. Using this result as the cut-off value, patients with an age of LUTS onset < 65 years and ≥65 years were assigned to the early- and late-onset group, respectively.

The age at LUTS onset (early-onset group, 55.3 ± 6.6 years; late-onset group, 73.9 ± 5.8 years; *p* < 0.001) and the age at OAB diagnosis (early-onset group, 68.7 ± 9.0 years; late-onset group, 78.1 ± 5.7 years; *p* < 0.001) were significantly higher in the late-onset group than that in the early-onset group. Overall, 18 patients (31.0%) in the early-onset group and 41 patients (58.6%) in the late-onset group demonstrated the capacity to produce equol (*p* = 0.002). In addition, the urinary equol concentration in the late-onset group was significantly higher than that in the early-onset group (*p* = 0.014) (Table 2).

No significant differences were observed in the general comorbidities between the two groups. No significant differences were observed between the two groups in the OABSS; however, PVR was significantly higher in the late-onset group than that in the early-onset group (early-onset group, 17.5 ± 29.0 mL; late-onset group, 32.5 ± 40.1 mL; *p* < 0.001) (Table 2).

### 3.4. Equol Production Capacity and Factors Associated with the Onset of OAB Based on the Time of Diagnosis

Table 3 presents the differences in various parameters based on the timing of LUTS onset and equol production.

In early-onset individuals, no significant differences were observed in equol production capacity in terms of age at diagnosis of OAB (62.2 ± 8.1 years vs. 64.4 ± 9.4 years, *p* = 0.400) or age of LUTS onset (55.1 ± 6.0 years vs. 55.4 ± 6.7 years, *p* = 0.866). However, hypertension was significantly more common in the P group than in the NP group (61.1% vs. 32.5%, *p* = 0.049), and chronic kidney disease was significantly less common (16.5% vs. 45.0%, *p* = 0.044) in early-onset individuals. Additionally, the P group had a lower urgency (OABSS Q3) and total OABSS than the NP group; however, these differences were not significant (Q3, *p* = 0.123; total OABSS, *p* = 0.127). Similarly, no significant differences were found in the severity of OAB between the two groups (*p* = 0.649) (Table 3). No significant differences were observed in early-onset individuals between the P and NP groups in terms of objective findings.

In late-onset individuals, no significant differences were observed between the P group and NP group in terms of age at OAB diagnosis (78.1 ± 5.7 years vs. 78.1 ± 6.0 years, *p* = 0.963) or age of LUTS onset (74.7 ± 6.0 years vs. 72.8 ± 5.5 years, *p* = 0.171). Similarly, no significant differences were observed between the two groups in terms of comorbidities, subjective symptoms, or objective findings (Table 3).

### 3.5. Correlation Between Urinary Equol Level and Patients’ Backgrounds and Urological Symptoms

Urinary equol concentration showed no correlation with patient characteristics or urological parameters, including subjective and objective measures. Although VV appeared to be mildly correlated with equol production (*p* = 0.063), the correlation was weak and positive (*r* = 0.211). However, Qmax and PVR did not correlate with urinary equol levels (Table 4).

### 3.6. Predictive Factors for Early-Onset LUTS

We performed univariate analyses to identify risk factors for early-onset LUTS. The results revealed that equol production was a significant predictor of early-onset LUTS (Table 5). Furthermore, we assessed whether equol production could serve as a predictor of early-onset LUTS using propensity score matching (Table 6 and Table 7) for 104 patients (52 patients in each cohort). The standardized mean differences for all characteristics were <0.02, indicating minimal baseline differences between the groups (Table 6). Equol non-producers were significantly more likely to have early-onset compared to equol producers (*p* = 0.014; OR, 1.930; 95% CI, 1.248–4.049) (Table 7).

## 4. Discussion

The menopausal transition is marked by fluctuating and ultimately declining estrogen levels, which can contribute to the emergence of LUTS, including urgency, frequency, and nocturia. These symptoms often begin during perimenopause and may worsen after menopause, significantly impairing quality of life. Despite the known role of estrogen in maintaining urogenital health, interindividual differences in symptom onset suggest that other modulatory factors—such as the ability to produce equol—may also influence susceptibility to LUTS [7,15].

This study investigated the relationship between equol production capacity and onset of LUTS, including OAB, in postmenopausal women. The results showed that patients capable of producing equol were significantly more likely to experience LUTS at a later age than non-producers. This association remained significant even after adjusting for potential confounders using propensity score matching, suggesting that non-production of equol may be an independent risk factor for early-onset LUTS. Additionally, equol production was not subjectively or objectively associated with OAB symptom severity. This suggests that equol may influence the timing of symptom onset rather than symptom intensity and may function more as a preventive or modulatory factor than a therapeutic one. Furthermore, considering the estrogenic activity of equol, our findings imply that it may play a protective role in bladder aging and serve as a potential biomarker or target for early intervention for OAB in postmenopausal women.

Our findings are consistent with a growing body of literature indicating that equol, a gut-derived metabolite of the soy isoflavone daidzein, exhibits estrogen-like activity primarily mediated through selective binding to estrogen receptor beta (ER-β) [16,17]. ER-β is widely expressed in the bladder, urethra, and pelvic floor tissues, and is thought to regulate tissue maintenance, neuromuscular function, and epithelial integrity [18]. These receptor-specific effects make equol a promising alternative to systemic hormone replacement therapy (HRT), which has shown mixed results and has safety concerns in the context of LUTS management.

Although several clinical trials have confirmed that equol supplementation improves menopausal symptoms, such as vasomotor instability and mood disturbances [19,20], its role in urological health remains underexplored. In this regard, the present study provides novel evidence demonstrating that endogenous equol production, rather than supplementation alone, is associated with the delayed onset of LUTS. This delay in symptom onset may reflect the cumulative long-term effects of equol on urogenital tissues, a hypothesis supported by previous findings based on its tissue-selective estrogenic actions. These results are particularly relevant in Asian populations, where equol-producing individuals are more prevalent compared to Western populations [17]. Consequently, these results raise important considerations regarding population-specific screening strategies and dietary recommendations tailored to the status of individual equol producers.

Furthermore, our findings suggest that equol production may influence the timing of LUTS onset, particularly during the menopausal transition when estrogen fluctuations begin to impact urogenital tissues. However, once structural or neurological degeneration has occurred, equol may have limited therapeutic efficacy. This interpretation is supported by epidemiological data showing increased OAB prevalence in individuals aged ≥40 years [21], and previous trials reporting greater symptom reduction in equol-producers receiving higher doses or more frequent administration during the perimenopausal period than equol non-producers [22]. These observations suggest that equol may be more valuable as a preventive biomarker or risk modifier than as a treatment for existing LUTS, and that its clinical utility may depend on individual metabolic capability and menopausal stage [19,22,23]. This distinction has important implications for the design of future intervention trials and patient selection in personalized treatment approaches. Additionally, these findings are consistent with previous observations stating that the menopausal transition is associated with multiple health concerns—including sleep disturbances, mood changes, and urogenital symptoms—that can severely impact quality of life [7]. While the present study focused on urological outcomes, future studies should explore whether the modulatory effects of equol extend to these broader domains of menopausal health. Identifying such modifiable factors could inform comprehensive strategies to support women’s well-being throughout midlife and beyond.

Several biologically plausible mechanisms could underlie the observed association between equol production and the delayed onset of LUTS without necessarily affecting the severity once dysfunction is present. Equol is a nonsteroidal, estrogen-like compound that selectively activates ER-β, which plays a crucial role in maintaining bladder and pelvic tissue homeostasis [16,17]. ER-β activation promotes anti-inflammatory responses, maintains collagen synthesis, and supports urothelial barrier integrity—functions that decline during menopause and contribute to LUTS [18,24,25]. Consequently, estrogen deficiency is known to accelerate bladder wall thinning, reduce elasticity, and impair detrusor contractility, all of which predispose to OAB symptoms [4,18,26,27]. Additionally, estrogen modulates neurotransmitter systems in the bladder, including muscarinic and purinergic receptors, which play key roles in bladder sensation and detrusor overactivity [18,28,29]. Thus, equol may buffer these degenerative processes by mimicking estrogen in ER-β-specific pathways.

Furthermore, recent studies support the existence of a gut–bladder axis, wherein the intestinal microbiota influences both equol production and systemic inflammation. For example, *Adlercreutzia equolifaciens*, an equol-producing bacterium, is less abundant in patients with metabolic liver disease, suggesting that equol plays a role in modulating inflammation [30]. Additionally, dietary isoflavones, the precursors of equol, alter the composition of the gut microbiota and reduce pro-inflammatory markers [31]. These findings highlight how the gut microbiota can impact systemic conditions, possibly affecting bladder health through inflammatory pathways. Therefore, equol producers may harbor more favorable microbiota profiles that contribute to reduced systemic and local inflammation, an increasingly recognized factor in the pathophysiology of LUTS. Accordingly, our results suggest that equol producer status could serve as a non-invasive biomarker for the early identification of postmenopausal women at an increased risk of early-onset LUTS. Moreover, urinary equol testing is feasible, cost-effective, and well tolerated, making it suitable for midlife risk stratification [16,17]. This opens the possibility of targeted prevention efforts long before clinical symptoms emerge.

Given the receptor specificity and favorable safety profile of equol [14,15], it may represent a safer alternative to systemic HRT, particularly for women at risk of estrogen-sensitive conditions. Consequently, future randomized controlled trials are needed to evaluate whether dietary or microbiota-based interventions—such as soy isoflavone supplementation or tailored probiotics—can enhance equol production and reduce LUTS risk, especially in non-producers [17,18,19,20]. Thus, our findings support a shift toward personalized preventive urology that integrates hormonal, nutritional, and microbial factors as modifiable determinants of bladder health in aging women. Taken together, our findings, integrated with prior research and a mechanistic understanding, highlight the potential of equol as a preventive, personalized tool in urological aging. This reinforces the clinical relevance of assessing equol status in midlife women and sets the stage for translational research into diet- and microbiota-based interventions.

Nonetheless, this study had some limitations. First, the cross-sectional design of the study precludes conclusions regarding causality. Although a significant association was observed between equol production and delayed onset of LUTS, prospective cohort studies are required to confirm this relationship. Second, equol production was assessed using a single urine measurement, which may not reflect long-term production owing to day-to-day variations in diet and microbiome composition. Consequently, repeated sampling, along with detailed dietary and lifestyle assessments, should be incorporated into future studies. Third, the sample size was relatively small and was drawn from a single institution, potentially introducing selection bias and limiting generalizability. Larger multicenter studies with ethnically and geographically diverse populations would enhance external validity. Fourth, the classification of patients into early- and late-onset groups was based on interviews conducted during the initial consultation. As this study relied on the self-reported recollection of LUTS onset, recall bias could not be ruled out. Additionally, the accuracy of the reported age at symptom onset may have been affected by the patients’ memory, potentially influencing group assignment. Thus, future studies may benefit from prospective symptom tracking to more precisely determine the onset timing. Finally, we did not assess the gut microbiome profile, which likely influences equol production and urological aging. Therefore, the integration of metagenomic, metabolomic, and inflammatory biomarkers would help elucidate mechanisms and identify new intervention targets. Furthermore, future trials should examine whether modifying equol status through dietary means, supplementation, or microbiome modulation can prevent or delay LUTS in postmenopausal women, especially in those identified as non-producers.

## 5. Conclusions

This study provides new evidence that equol production is significantly associated with the delayed onset of LUTS in postmenopausal women, suggesting that equol plays a protective role in bladder aging. Although equol did not appear to influence the severity of symptoms once it emerged, its link to onset timing supports its utility as a preventive biomarker and modification target.

These findings highlight the importance of individualized prevention strategies in urology, particularly those that integrate nutritional, hormonal, and microbiome-related factors. Further longitudinal and interventional studies are warranted to confirm these associations and guide future clinical applications.

## Figures and Tables

**Table 1:** Patients’ background and urological parameters.

	Entire	P Group	NP Group	*p* Value
Number of patients (%)	128 (100)	59 (46.1)	69 (53.9)	-
Age (years)	71.6 ± 10.3	73.2 ± 9.8	70.2 ± 10.6	0.094
Age of onset of LUTS (years)	65.5 ± 11.2	68.7 ± 10.9	62.7 ± 10.7	0.002
Urinary equol concentration (μmol/L)	11.7 ± 38.2	37.4 ± 176.4	0.2 ± 0.3	<0.001
Hypertension (%)	64 (50.0)	36 (61.0)	28 (40.6)	0.033
Diabetes mellitus (%)	23 (18.0)	13 (22.0)	10 (14.5)	0.356
Dyslipidemia (%)	34 (26.6)	17 (28.8)	17 (24.6)	0.689
Chronic kidney disease (%)	47 (36.7)	18 (30.5)	29 (42.0)	0.201
Subjective symptoms (OABSS)				
Q1. Daytime frequency	1.0 ± 0.6	1.0 ± 0.6	1.0 ± 0.6	0.930
Q2. Nocturia	1.9 ± 1.0	1.9 ± 1.0	1.9 ± 1.1	0.958
Q3. Urgency	3.3 ± 1.1	3.2 ± 1.1	3.5 ± 1.1	0.104
Q4. Urgency incontinence	2.2 ± 1.8	2.08 ± 1.7	2.2 ± 1.9	0.711
Total OABSS	8.4 ± 3.0	8.2 ± 3.0	8.6 ± 3.1	0.429
Severity of OAB				0.845
Mild (%)	13 (15.5)	10 (16.9)	14 (20.3)	
Moderate (%)	42 (50.0)	39 (66.1)	42 (60.9)	
Severe (%)	29 (34.5)	10 (16.9)	13 (18.8)	
Objective findings				
Voided volume (mL)	219.9 ± 131.0	201.4 ± 118.8	236.7 ± 140.4	0.231
Maximum flow rate (mL/s)	21.0 ± 12.4	20.3 ± 12.3	21.7 ± 12.6	0.610
Post-void residual urine (mL)	35.6 ± 36.0	23.4 ± 33.2	27.7 ± 38.8	0.577

P, equol-producing; NP, equol-non-producing; LUTS, lower urinary tract symptom; OABSS, overactive bladder symptom score; OAB, overactive bladder.

**Table 2:** Differences in patients’ backgrounds based on onset time.

	Early-Onset	Late-Onset	*p* Value
Number of patients (%)	58 (45.3)	70 (54.7)	-
Equol producing (%)	18 (31.0)	41 (58.6)	0.002
Age at diagnosis of OAB (years)	68.7 ± 9.0	78.1 ± 5.7	<0.001
Age of onset of LUTS (years)	55.3 ± 6.6	73.9 ± 5.8	<0.001
Urinary equol concentration (μmol/L)	3.6 ± 8.5	29.3 ± 163.8	0.014
Hypertension (%)	24 (41.4)	40 (57.1)	0.110
Diabetes mellitus (%)	11 (19.0)	12 (17.1)	0.820
Dyslipidemia (%)	12 (20.7)	22 (31.4)	0.228
Chronic kidney disease (%)	21 (36.2)	26 (37.1)	>0.999
Subjective symptoms (OABSS)			
Q1. Daytime frequency	1.0 ± 0.6	0.9 ± 0.6	0.342
Q2. Nocturia	1.7 ± 1.1	2.1 ± 1.0	0.052
Q3. Urgency	3.3 ± 1.1	3.3 ± 1.2	0.996
Q4. Urgency incontinence	1.9 ± 1.8	2.4 ± 1.8	0.148
Total OABSS	8.0 ± 2.9	8.7 ± 3.1	0.175
Severity of OAB			0.159
Mild (%)	14 (24.1)	10 (14.3)	
Moderate (%)	37 (63.8)	44 (62.9)	
Severe (%)	7 (12.1)	16 (22.9)	
Objective findings			
Voided volume (mL)	222.3 ± 136.7	217.8 ± 127.5	0.880
Maximum flow rate (mL/s)	21.2 ± 11.7	20.8 ± 13.1	0.906
Post-void residual urine (mL)	17.5 ± 29.0	32.5 ± 40.1	0.048

LUTS, lower urinary tract symptom; OAB, overactive bladder; OABSS, overactive bladder symptom score.

**Table 3:** Differences in various parameters based on timing of urinary symptoms onset and equol production ability.

	Early-Onset			Late-Onset		
	P Group	NP Group	*p* Value	P Group	NP Group	*p* Value
Number of patients (%)	18 (31.0)	40 (69.0)	-	41 (58.6)	29 (41.4)	-
Age (years)	62.2 ± 8.1	64.4 ± 9.4	0.400	78.1 ± 5.7	78.1 ± 6.0	0.963
Age of onset of LUTS (years)	55.1 ± 6.0	55.4 ± 6.7	0.866	74.7 ± 6.0	72.8 ± 5.5	0.171
Urinary equol concentration (μmol/L)	10.7 ± 12.2	0.2 ± 0.3	<0.001	49.2 ± 211.2	0.2 ± 0.2	<0.001
Hypertension (%)	11 (61.1)	13 (32.5)	0.049	25 (61.0)	15 (51.7)	0.472
Diabetes mellitus (%)	5 (27.8)	6 (15.0)	0.290	8 (19.5)	4 (13.8)	0.749
Dyslipidemia (%)	5 (27.8)	7 (17.5)	0.486	12 (29.3)	10 (34.5)	0.794
Chronic kidney disease (%)	3 (16.7)	18 (45.0)	0.044	15 (36.6)	11 (37.9)	1.000
Subjective symptoms (OABSS)						
Q1. Daytime frequency	1.1 ± 0.5	1.0 ± 0.6	0.750	0.9 ± 0.7	0.9 ± 0.6	0.839
Q2. Nocturia	1.6 ± 1.0	1.8 ± 1.1	0.544	2.1 ± 1.0	2.1 ± 1.0	0.788
Q3. Urgency	3.0 ± 0.9	3.5 ± 1.1	0.123	3.2 ± 1.2	3.5 ± 1.1	0.360
Q4. Urgency incontinence	1.4 ± 1.4	2.1 ± 1.9	0.201	2.4 ± 1.7	2.3 ± 1.9	0.961
Total OABSS	7.1 ± 2.0	8.4 ± 3.2	0.127	8.6 ± 3.3	8.9 ± 2.9	0.741
Severity of OAB			0.649			1.000
Mild (%)	4 (22.2)	10 (25.0)		6 (14.6)	4 (13.8)	
Moderate (%)	13 (72.2)	24 (60.0)		26 (63.4)	18 (62.1)	
Severe (%)	1 (5.6)	6 (15.0)		9 (22.0)	7 (24.1)	
Objective findings						
Voided volume (mL)	193.1 ± 100.2	236.3 ± 151.0	0.376	205.1 ± 128.2	237.2 ± 127.8	0.426
Maximum flow rate (mL/s)	16.8 ± 4.9	23.3 ± 13.4	0.089	21.9 ± 14.2	43.4 ± 48.2	0.467
Post-void residual volume (mL)	17.5 ± 32.4	17.5 ± 27.9	0.819	26.1 ± 33.8	43.4 ± 48.2	0.147

OABSS, overactive bladder symptom score.

**Table 4:** Correlation between urinary equol concentration levels and urological parameters.

	*r*	95% CI	*p* Value
Age (years)	0.062	−0.116–0.236	0.495
Age of onset of LUTS (years)	0.043	−0.135–0.217	0.637
Subjective symptoms (OABSS)			
Q1. Daytime frequency	−0.006	−0.183–0.170	0.943
Q2. Nocturia	0.109	−0.069–0.280	0.228
Q3. Urgency	0.137	−0.041–0.306	0.130
Q4. Urgency incontinence	−0.088	−0.260–0.090	0.332
Total OABSS	0.035	−0.142–0.210	0.696
Objective findings			
Voided volume (mL)	0.211	−0.012–0.414	0.063
Maximum flow rate (mL/s)	0.106	−0.106–0.308	0.327
Post-void residual urine (mL)	−0.007	−0.215–0.202	0.951

CI, confidence interval; LUTS, lower urinary tract symptom; OABSS, overactive bladder symptom score.

**Table 5:** Risk factors for early-onset of LUTS.

	Univariate Analysis		
	OR	95% CI	*p* Value
Hypertension	1.890	0.933–3.820	0.077
Diabetes mellitus	0.884	0.358–2.180	0.789
Dyslipidemia	1.760	0.781–3.950	0.173
Chronic kidney disease	1.040	0.506–2.140	0.913
Equol production	3.140	1.610–6.530	0.002

OR, odds ratio; CI, confidence interval.

**Table 6:** Baseline characteristic data of age of LUTS onset after propensity score matching.

	Early-Onset	Late-Onset	*p* Value	Standardized Mean Differences
Hypertension (%)	24 (46.2)	23 (44.4)	>0.999	0.019
Diabetes mellitus (%)	6 (11.5)	6 (11.5)	>0.999	0.011
Dyslipidemia (%)	12 (23.1)	13 (25.0)	>0.999	0.017
Chronic kidney disease (%)	18 (34.6)	19 (36.5)	>0.999	0.015

LUTS, lower urinary tract symptom.

**Table 7:** Differences in equol production according to age of LUTS onset.

	Early-Onset N = 52	Late-Onset N = 52	*p* Value
Equol production; positive (%)	16 (30.8)	24 (46.2)	0.014

LUTS, lower urinary tract symptom.

## Data Availability

The data supporting this study are available from the corresponding author upon request. The data are not publicly accessible owing to privacy and ethical considerations.

## Abbreviations

The following abbreviations are used in this manuscript:

OAB	overactive bladder
OABSS	overactive bladder symptom score
LUTS	lower urinary tract symptom
ERs	estrogen receptors
Q	question
P	equol-producing
NP	equol-non-producing
VV	voided volume
Qmax	maximum flow rate
PVR	post-void residual urine volume
ELISA	enzyme-linked immunosorbent assay
ER-β	estrogen receptor beta
HRT	hormone replacement therapy

## References

[1] Abrams P., Cardozo L., Fall M., Griffiths D., Rosier P., Ulmsten U., van Kerrebroeck P., Victor A., Wein A., Standardisation Sub-Committee of the International Continence Society (2002). The standardisation of terminology of lower urinary tract function: Report from the standardisation sub-committee of the international continence society. Neurourol. Urodyn..

[2] Qudah S., Abufaraj M., Farah R., Almazeedi A., Ababneh A., Alnabulsi M., Qatawneh A., Hyassat D., Ajlouni K. (2024). The prevalence of overactive bladder and its impact on the quality of life: A cross-sectional study. Arab J. Urol..

[3] Mitsui T., Sekido N., Masumori N., Haga N., Omae K., Saito M., Kubota Y., Sakakibara R., Yoshida M., Takahashi S. (2024). Prevalence and impact on daily life of lower urinary tract symptoms in Japan: Results of the 2023 Japan Community Health Survey (JaCS 2023). Int. J. Urol..

[4] Angelou K., Grigoriadis T., Diakosavvas M., Zacharakis D., Athanasiou S. (2020). The genitourinary syndrome of menopause: An overview of the recent data. Cureus.

[5] Ramezani Tehrani F., Mousavi M., Saei Ghare Naz M., Noroozzadeh M., Azizi F., Farahmand M. (2025). Endogenous estrogen exposure and hypertension risk; A population-based cohort study with about 2 decades of follow-up. J. Clin. Endocrinol. Metab..

[6] Baruch Y., Torella M., De Bastiani S., Meschia M., Candiani M., Colacurci N., Salvatore S. (2023). Pre- versus post-Menopausal Onset of overactive bladder and the Response to Vaginal estrogen Therapy: A Prospective Study. Medicina.

[7] Gold E.B. (2011). The timing of the age at which natural menopause occurs. Obstet. Gynecol. Clin. N. Am..

[8] Gong Y., Lv J., Pang X., Zhang S., Zhang G., Liu L., Wang Y., Li C. (2023). Advances in the metabolic mechanism and functional characteristics of equol. Foods.

[9] Zhang W., Zhang Y., Li J., Tang J., Wu J., Xie Z., Huang X., Tao S., Xue T. (2024). Identification of metabolites from the gut microbiota in hypertension via network pharmacology and molecular docking. Bioresour. Bioprocess..

[10] Cheong S.H., Furuhashi K., Ito K., Nagaoka M., Yonezawa T., Miura Y., Yagasaki K. (2014). Antihyperglycemic effect of equol, a daidzein derivative, in cultured L6 myocytes and ob/ob mice. Mol. Nutr. Food Res..

[11] Yoshikata R., Myint K.Z.Y., Ohta H., Ishigaki Y. (2021). Effects of an equol-containing supplement on advanced glycation end products, visceral fat and climacteric symptoms in postmenopausal women: A randomized controlled trial. PLoS ONE.

[12] Homma Y., Yoshida M., Seki N., Yokoyama O., Kakizaki H., Gotoh M., Yamanishi T., Yamaguchi O., Takeda M., Nishizawa O. (2006). Symptom assessment tool for overactive bladder syndrome-overactive bladder symptom score. Urology.

[13] Igase M., Igase K., Tabara Y., Ohyagi Y., Kohara K. (2017). Cross-sectional study of equol producer status and cognitive impairment in older adults. Geriatr. Gerontol. Int..

[14] Takeda T., Shiina M., Chiba Y. (2018). Effectiveness of natural S-equol supplement for premenstrual symptoms: Protocol of a randomised, double-blind, placebo-controlled trial. BMJ Open.

[15] Setchell K.D.R., Clerici C. (2010). Equol: History, chemistry, and formation. J. Nutr..

[16] Setchell K.D.R., Brown N.M., Lydeking-Olsen E. (2002). The clinical importance of the metabolite equol—A clue to the effectiveness of soy and its isoflavones. J. Nutr..

[17] Yuan J.P., Wang J.H., Liu X. (2007). Metabolism of dietary soy isoflavones to equol by human intestinal microflora—Implications for health. Mol. Nutr. Food Res..

[18] Robinson D., Cardozo L.D. (2003). The role of estrogens in female lower urinary tract dysfunction. Urology.

[19] Ishiwata N., Melby M.K., Mizuno S., Watanabe S. (2009). New equol supplement for relieving menopausal symptoms: Randomized, placebo-controlled trial of Japanese women. Menopause.

[20] Uesugi S., Watanabe S., Ishiwata N., Uehara M., Ouchi K. (2004). Effects of isoflavone supplements on bone metabolic markers and climacteric symptoms in Japanese women. BioFactors.

[21] Temml C., Heidler S., Ponholzer A., Madersbacher S. (2005). Prevalence of the overactive bladder syndrome by applying the International Continence Society definition. Eur. Urol..

[22] Crawford S.L., Jackson E.A., Churchill L., Lampe J.W., Leung K., Ockene J.K. (2013). Impact of dose, frequency of administration, and equol production on efficacy of isoflavones for menopausal hot flashes: A pilot randomized trial. Menopause.

[23] Wu J., Oka J., Ezaki J., Ohtomo T., Ueno T., Uchiyama S., Toda T., Uehara M., Ishimi Y. (2007). Possible role of equol status in the effects of isoflavone on bone and fat mass in postmenopausal Japanese women: A double-blind, randomized, controlled trial. Menopause.

[24] Fan W., Ding C., Liu S., Gao X., Shen X., De Boevre M., Gao Z., Li M., Zhang S., Miao Y. (2022). Estrogen receptor β activation inhibits colitis by promoting NLRP6-mediated autophagy. Cell Rep..

[25] Cvoro A., Tatomer D., Tee M.K., Zogovic T., Harris H.A., Leitman D.C. (2008). Selective estrogen receptor-β agonists repress transcription of proinflammatory genes. J. Immunol..

[26] Yang X., Li Y.Z., Mao Z., Gu P., Shang M. (2009). Effects of estrogen and tibolone on bladder histology and estrogen receptors in rats. Chin. Med. J..

[27] Sánchez-Ortiz R.F., Wang Z., Menon C., DiSanto M.E., Wein A.J., Chacko S. (2001). Estrogen modulates the expression of myosin heavy chain in detrusor smooth muscle. Am. J. Physiol. Cell Physiol..

[28] Russo E., Misasi G., Montt-Guevara M.M., Giannini A., Simoncini T. (2023). Effects of ospemifene on overactive bladder in postmenopausal women with vulvovaginal atrophy. Climacteric.

[29] Setchell K.D.R., Clerici C., Lephart E.D., Cole S.J., Heenan C., Castellani D., Wolfe B.E., Nechemias-Zimmer L., Brown N.M., Lund T.D. (2005). S-equol, a potent ligand for estrogen receptor β, is the exclusive enantiomeric form of the soy isoflavone metabolite produced by human intestinal bacterial flora. Am. J. Clin. Nutr..

[30] Oñate F.P., Chamignon C., Burz S.D., Lapaque N., Monnoye M., Philippe C., Bredel M., Chêne L., Farin W., Paillarse J.M. (2023). Adlercreutzia equolifaciens is an anti-inflammatory commensal bacterium with decreased abundance in gut microbiota of patients with metabolic liver disease. Int. J. Mol. Sci..

[31] Ghimire S., Cady N.M., Lehman P., Peterson S.R., Shahi S.K., Rashid F., Giri S., Mangalam A.K. (2022). Dietary isoflavones alter gut microbiota and lipopolysaccharide biosynthesis to reduce inflammation. Gut Microbes.

